# Coincidence of potato *CONSTANS* (*StCOL1*) expression and light cannot explain night‐break repression of tuberization

**DOI:** 10.1111/ppl.12885

**Published:** 2018-12-21

**Authors:** Faline D. M. Plantenga, Ep Heuvelink, Juriaan A. Rienstra, Richard G. F. Visser, Christian W. B. Bachem, Leo F. M. Marcelis

**Affiliations:** ^1^ Horticulture and Product Physiology Wageningen University & Research Wageningen The Netherlands; ^2^ Plant Breeding Wageningen University & Research Wageningen The Netherlands

## Abstract

In the obligate short‐day potato *Solanum tuberosum* group Andigena (*Solanum andigena*), short days, or actually long nights, induce tuberization. Applying a night break in the middle of this long night represses tuberization. However, it is not yet understood how this repression takes place. We suggest a coincidence model, similar to the model explaining photoperiodic flowering in Arabidopsis. We hypothesize that potato *CONSTANS* (*StCOL1*), expressed in the night of a short day, is stabilized by the light of the night break. This allows for StCOL1 to repress tuberization through induction of *StSP5G*, which represses the tuberization signal *StSP6A*. We grew *S. andigena* plants in short days, with night breaks applied at different time points during the dark period, either coinciding with *StCOL1* expression or not. StCOL1 protein presence, *StCOL1* expression and expression of downstream targets *StSP5G* and *StSP6A* were measured during a 24‐h time course. Our results show that a night break applied during peak *StCOL1* expression is unable to delay tuberization, while coincidence with low or no *StCOL1* expression leads to severely repressed tuberization. These results imply that coincidence between *StCOL1* expression and light does not explain why a night break represses tuberization in short days. Furthermore, stable StCOL1 did not always induce *StSP5G*, and upregulated *StSP5G* did not always lead to fully repressed *StSP6A*. Our findings suggest there is a yet unknown level of control between *StCOL1*, *StSP5G* and *StSP6A* expression, which determines whether a plant tuberizes.

**Abbreviations**
HA: hemagglutinin
LD: long day
NB: night break
PHYB: phytochrome B
RT: room temperature
SD: short day
SDS: sodium dodecyl sulfate
StCOL1: StCONSTANS-like 1
ZT: Zeitgeber time

## Introduction

Globally, potato (*Solanum tuberosum*) is the third most consumed food crop by humans (http://cipotato.org/potato). Gaining an extensive understanding on tuberization (tuber formation) can give growers the tools that are needed to optimize growth and tuber yield.

Light strongly influences potato plant development and the day‐length is a key factor controlling tuberization (Prat 2010). Shorter day‐lengths promote tuberization in potatoes (Garner and Allard 1923, Rodríguez‐Falcón et al. 2006). Although tuberization in all potato varieties is induced in short days, not all varieties require short days to tuberize (Driver and Hawkes 1943). *Solanum tuberosum* group Andigena (*Solanum andigena*) is a variety that originated in the Andes, and is used as a model potato plant for photoperiod studies, because of its strict short‐day requirement for tuberization (<12 h light; Ewing and Struik 1992, Jackson 1999, Hannapel 2007). It is supposed that not the length of the light period, but the length of the dark period is critical for tuberization (Jackson 1999), therefore not short days but long nights induce tuberization. A night break, i.e. a short period of white or red light in the middle of the night, can be applied during a long night to repress tuberization (Batutis and Ewing 1982, Jackson et al. 1996, Macháčková et al. 1998). A night break not only represses short‐day tuberization, but also short‐day flowering in species like Chrysanthemum (Borthwick and Cathey 1962, Horridge and Cockshull 1989, Higuchi et al. 2012). Furthermore, night breaks given in the long nights of a short day can induce flowering in long‐day plants like wheat (Pearce et al. 2017). Although a lot of research has been done on night breaks, it is not fully understood how a night break regulates tuberization. It has been suggested that tuberization is repressed by a night break because the night break divides a long night into two short nights (Jackson 1999). It has also been proposed that the red/far‐red photoreceptor PHYTOCHROME B (PHYB) plays a role in the control of a night break. Applying a period of red light in the middle of a long night and subsequently applying far‐red light reversed repression on tuberization imposed by a night break (Batutis and Ewing 1982). However, when these experiments were performed, the molecular control behind tuberization was not yet known. With the help of new molecular knowledge of tuberization regulation, we may be able to unravel the functioning of a night break.

The genes controlling tuberization are conserved even in species that do not form tubers, including *Arabidopsis thaliana*, *Chrysanthemum lavandulifolium* and *Oryza sativa* (rice). Orthologs of these genes are responsible for photoperiodic control of flowering (Martínez‐García et al. 2002, Tsuji et al. 2011, Andrés and Coupland 2012, Fu et al. 2014). In Arabidopsis, long‐day flowering is induced through the coincidence of gene expression and light, which only allow induction of *FLOWERING LOCUS T* (*FT*) under long‐day conditions (Andrés and Coupland 2012). *FT* encodes for the mobile florigen, which moves from the leaves to the shoot apical meristem where it induces flowering. The molecular control of photoperiodic flowering is reviewed in the studies of Imaizumi and Kay (2006), Andrés and Coupland (2012) and Song et al. (2014): *CONSTANS* (*CO*) expression is controlled by circadian clock components, which at the end of a long day form a complex that degrades CYCLING DOF FACTOR (CDF), an inhibitor of *CO*, allowing for *CO* expression at the end of a long day. In short days, CDF is not degraded by this complex, only allowing *CO* to be expressed in the night. Post‐translational control of CO by light further determines whether flowering is induced. In the light, photoreceptors PHYTOCHROME A (PHYA; red/far‐red light) and CRYPTOCHROME (CRY; blue light) reduce the activity of a complex that degrades CO. In short days, *CO* is only expressed in the night where CO is degraded. In long days, *CO* expression takes place in the light where CO is not degraded, leading to the induction of *FT* and the initiation of flowering. A similar coincidence of light and internal gene expression also controls flowering time in the short‐day plant rice. An ortholog of *CO* in rice called *HEADING DATE 1* (*Hd1*), induces a rice *FT*, *Hd3a*, in short days, but represses it in long days (reviewed by Tsuji et al. 2011, Shrestha et al. 2014). Whether *Hd1* expression leads to induced or repressed *Hd3a* and thus to flowering was suggested to depend on the exposure of the Hd1 protein to light (active PHYB) (Ishikawa et al. 2011). In short days, the Hd1 would not be exposed to light, allowing for induction of *Hd3a* and flowering, while in long days Hd1 would be exposed to light, leading to repressed *Hd3a* and inhibited flowering.

**Figure 1:**
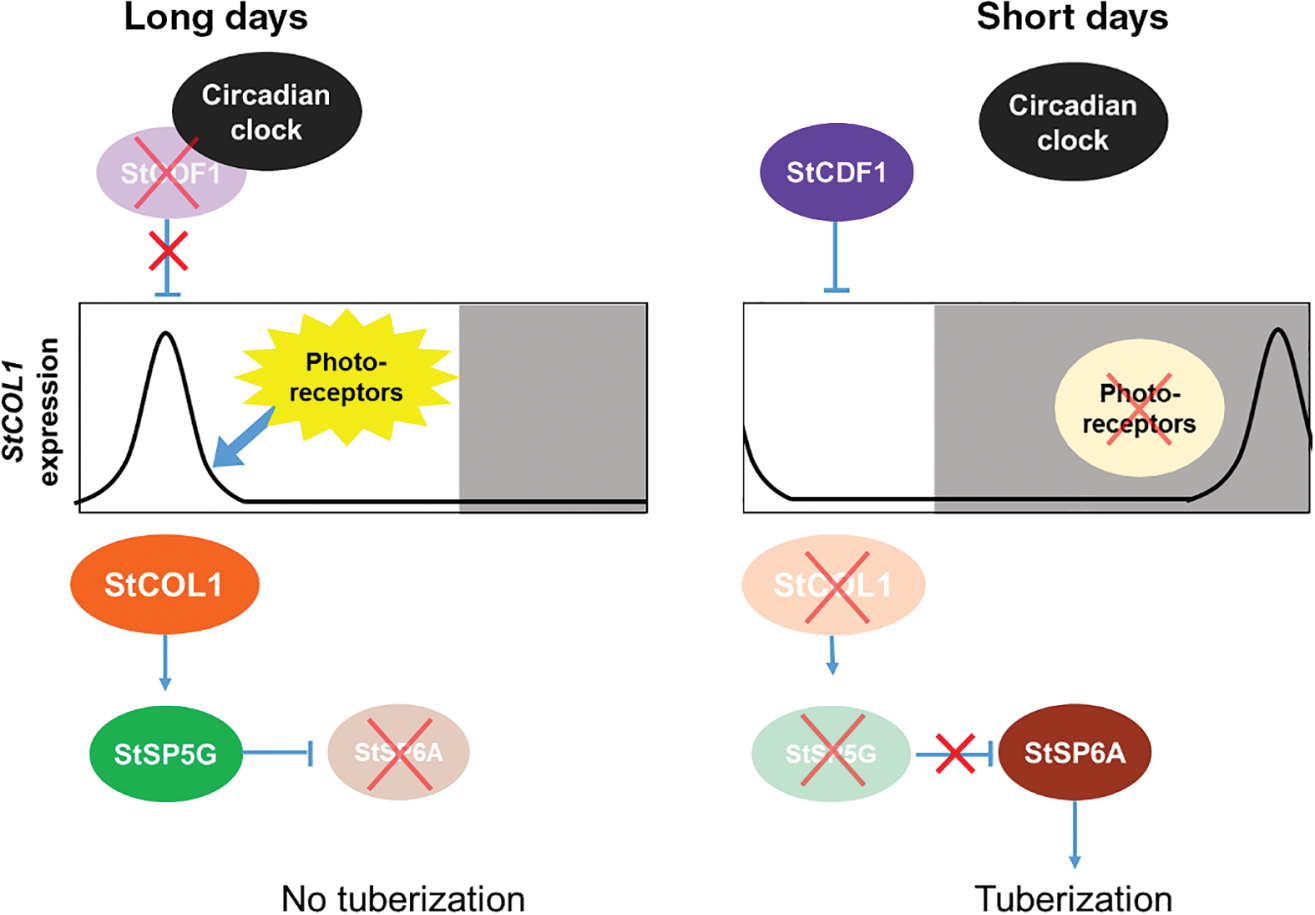
Photoperiodic control on potato tuberization based on a coincidence model of light and *StCOL1* expression (adapted from Navarro et al. 2015). In long days, circadian clock proteins interact with a repressor of *StCOL1* expression called StCDF1, which leads to its degradation. Due to the degradation of this repressor, *StCOL1* expression can take place in the light period. In the light, photoreceptors (PHYB) are activated and StCOL1 protein is stabilized. StCOL1 induces *StSP5G*, which leads to the repression of *StSP6A*, and in turn to repression of tuberization. In short days, interaction between circadian clock proteins and StCDF1 is not expected to take place, allowing for StCDF1 to repress *StCOL1* expression during the light period. Although *StCOL1* is expressed in the dark period, the photoreceptors are not active in the dark and cannot stabilize StCOL1 protein, which leads to its degradation. Without stabilized StCOL1, *StSP5G* is not expressed, leading to accumulation of StSP6A and the induction of tuberization.

Although potato tuberization is a short‐day process, it shares a similar control mechanism with flowering in Arabidopsis and, in part, with rice. In long days, circadian clock genes degrade the potato ortholog of CDF, StCDF1, which releases repression on the potato ortholog of *CO* (PGSC0003DMT400026065, named *StCONSTANS‐like1*, *StCOL1*; Abelenda et al. 2016) (Kloosterman et al. 2013). This allows for StCOL1 to induce an *FT*‐like repressor *SELF‐PRUNING 5G* (*StSP5G*), which represses the *FT*‐like tuberization factor *SELF‐PRUNING 6A* (*StSP6A*; Navarro et al. 2011, González‐Schain et al. 2012, Abelenda et al. 2016). In short days, little to no StCOL1 protein is present to induce *StSP5G*, allowing *StSP6A* expression in the leaves, after which StSP6A travels down to the stolons and induces tuberization (Navarro et al. 2011, Abelenda et al. 2016). Surprisingly, in both long and short days, *StCOL1* is expressed (González‐Schain et al. 2012, Abelenda et al. 2016), yet the StCOL1 protein is only present in long days. Abelenda et al. (2016) proposed that StCOL1 is only present in long days, because peak *StCOL1* expression coincides with the light in long days, while peak *StCOL1* expression in short days is shifted back and occurs at the end of the dark period. Song et al. (2014) also suggested that occurrence of peak expression in light is crucial for the coincidence model. In long days, coincidence between *StCOL1* peak expression and light would stabilize StCOL1 and repress tuberization, while in short days, *StCOL1* peak expression would coincide with the dark and StCOL1 would be degraded. A simplified model explaining the molecular control behind potato tuberization and the proposed coincidence model is described in Fig. 1.

As is the case in Arabidopsis, the stabilization of StCOL1 in potato is also controlled by photoreceptors. PHYB exists in an active and inactive form; red light (660 nm) activates PHYB and far‐red light (730 nm) inactivates it (Casal 2013). In daylight, which contains more red than far‐red light, the active form of PHYB is more prevalent. Red and far‐red light conversion to the active and inactive forms happens almost instantaneously. Active PHYB can also relax into inactive PHYB in the absence of light, but this dark reversion is a relatively slow process (Fankhauser 2001, Medzihradszky et al. 2013). In potato, active PHYB is responsible for stabilization of StCOL1 in the light (Abelenda et al. 2016).

This knowledge on the coincidence model in potato tuberization and involvement of PHYB may explain the molecular basis of a night break. PHYB regulated stabilization of StCOL1 in the light of a night break could lead to repression of tuberization in short days. However, *StCOL1* expression in a short day only peaks at the end of the night, and is low in the middle of the night when a night break is generally applied (González‐Schain et al. 2012). This would make StCOL1 stabilization in the light of a night break impossible. However, relaxation of PHYB to its inactive form may take hours (Ruddat et al. 1997, Fankhauser 2001). Therefore, PHYB may be activated in the light of a night break and remain active long enough to stabilize StCOL1 when *StCOL1* expression peaks at the end of the night. We hypothesize that the light applied during a night break represses tuberization by stabilizing StCOL1. StCOL1 can then induce *StSP5G*, which represses *StSP6A* and tuberization.

We aim to discover if night‐break repressed tuberization can be explained by light‐mediated stabilization of StCOL1 in the night. To find out whether this is the case, we grew *S. andigena* plants in short days with night breaks, which were applied at different moments during the night and determined if tuberization was repressed. The night breaks coincided with different levels of *StCOL1* expression, which was measured during a 24‐hour time course. Also, StCOL1 protein presence was measured, to see when StCOL1 was degraded or stabilized in the night. Furthermore, the expression of *StSP5G* and *StSP6A* was measured, to determine the effect of the different night breaks on downstream targets of StCOL1.

## Materials and methods

### Plant materials and growth conditions

A tetraploid, short‐day tuberizing *S. andigena* was used. A wild‐type and a transgenic line overexpressing *StCOL1* with a hemagglutinin (HA) tag (*35S::StCOL1‐HA*) in an *S. andigena* background were used for later protein detection by western blot. The transformation was done as described in Navarro et al. (2011) and the cloning of the genes and promotors was done as described in Abelenda et al. (2016).

The plants were propagated in vitro on MS20 medium (Murashige and Skoog 1962) and grown at 24°C in long days (16/8 h light/dark) in fluorescent light with a photosynthetic photon flux density of 200 μmol m^−2^ s^−1^. The plantlets from tissue culture were transplanted to square pots (7 × 7 × 8 cm) that were filled with a clay‐peat mixture and placed in two climate chambers. All plants were watered manually and liquid fertilizer was given once per week [Hydro Substrafeed Yara International, Oslo, Norway: 1.2 mM NH_4_^+^, 7.2 mM K^+^, 4.0 mM Ca^2+^, 1.82 mM Mg^2+^, 12.4 mM NO_3_^−^, 3.32 mM SO_4_^2−^, 10 mM P, 35 μM Fe^3+^, 8.0 μM Mn^2+^, 5.0 μM Zn^2+^, 20 μM B, 0.5 μM Cu^2+^, 0.5 μM MoO_4_^2−^, with an EC (electrical conductivity) of 2.0 dS m^−1^ and pH of 5.5]. The climate chambers were set at 20°C day and night, with a relative humidity of 70%. The plants were illuminated by red and white LEDs [light‐emitting diodes; GreenPower LED production module 120 cm DeepRedWhite‐2012, PSS: 0.88 (phytochrome stationary state; ‘1’ refers to only active PHYB and ‘0’ refers to only inactive PHYB); Philips, Eindhoven, the Netherlands]. LED height was adjusted every 2 weeks to maintain the required intensity (see experimental set‐up). The light intensity was measured at the top of the plant canopy with a quantum sensor (LI‐190SB quantum sensor, LI‐1400 Datalogger, LI‐COR Biosciences, Lincoln, NE). Plants were rotated three times a week to ensure uniform light distribution and all side‐shoots were removed from the plants.

### Experimental set‐up and measurements

**Figure 2:**
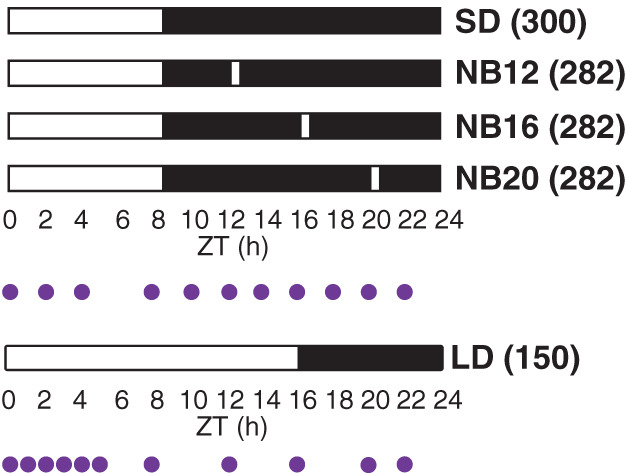
Schematic overview of light treatments with or without a night break. *Solanum andigena* potato plants were grown in five LED light treatments. A short day (SD), three short days with either an early night break (NB12), a regular night break (NB16) or a late night break (NB20) and one long day (LD). ZT, Zeitgeber time (hours after light stimulation). The white bar indicates light and the black bar indicates darkness. The numbers behind the treatments between parentheses show the light intensity in μmol m^−2^ s^−1^. In the night‐break treatments, the light given during the light period and the night break had the same intensity and color (red/white). All treatments received the same daily light sum (light intensity × hours of light per day). The purple dots show time points at which leaf material was sampled for gene expression analysis and protein detection.

Plants were grown in five climate chamber compartments, each containing a different light treatment. Light‐treatments included: (1) SD: a short‐day control treatment with 8/16 h light/dark, (2) NB12: a short day with a 30‐min night break early in the dark period (ZT(12); Zeitgeber time, hours after the start of light period), (3) NB16: a short day with a 30‐min night break in the middle of the night (ZT(16)), (4) NB20: a short day with a 30‐min night break towards the end of the dark period (ZT(20)) and (5) LD: a long‐day control treatment with 16/8 h light/dark. The night breaks were given every night, from the moment the plants were placed in the climate chamber until harvest. The light given during the night breaks was identical to the light given during the day and consisted of red and white LEDs. The treatments were divided over two climate chambers; one climate chamber was divided into four compartments with short‐day treatments and the other climate chamber contained the long‐day control treatment. Thirty‐three plantlets of the wild‐type and 33 plantlets of the *35S::StCOL1‐HA* line were placed in each light compartment. The light intensity applied was 300 μmol m^−2^ s^−1^ (short day), 282 μmol m^−2^ s^−1^ (short‐day + night‐break treatments) or 150 μmol m^−2^ s^−1^ (long day) (Fig. 2). In the night‐break treatments, the light intensity applied during the light period and during the night breaks was the same. The daily light integral of each treatment was the same (8.64 mol m^−2^ day^−1^). Four weeks after transplanting, plants were sampled for gene expression analysis and protein detection at 11 time points during a 24‐h cycle (Fig. 2). Of each plant, the fifth leaf from the shoot apex was sampled. Three plants were sampled per time point per treatment and each sample contained two leaflets. Samples were directly frozen in liquid nitrogen and stored at −80°C. After sampling, 10 plants were re‐potted to 17 cm Ø pots and maintained for four additional weeks. During this time, plants were examined for tuberization and flower bud appearance, three times a week (biological replicates n = 10). Destructive measurements were done after 8 weeks and included dry weights of stem, leaf and tubers, and the number of tubers.

### Gene expression and protein detection

Gene expression was determined using reverse transcription quantitative polymerase chain reaction (qPCR). For RNA extraction, the frozen plant material was ground to powder with a ball mill. Approximately 25 mg of plant sample was placed in a tube, to which 1.2 ml of TRIzol reagent was added (Invitrogen, Thermo Fisher, Waltham, MA). After mixing and incubating at room temperature (RT) for 5 min, samples were centrifuged at maximum speed at RT in a table‐top centrifuge. One milliliter of the supernatant was transferred, to which 250 μl chloroform was added and shaken. After centrifugation, 450 μl of the aqueous phase was mixed with 1 volume of isopropanol and samples were incubated at RT for 20 min. After centrifugation, the pellet was washed with 70% ethanol and dissolved in Milli‐Q water. Several RNA quality steps were performed (Taylor 2010); the integrity of the RNA was inspected by gel electrophoresis and quality of the RNA was tested by spectrophotometry (NanoDrop, DS11, DeNovix, Wilmington, DE). RNA integrity was checked on agarose gel, for all samples clear rRNA bands were visible (data not shown). One microgram of RNA was used for DNase treatment using RQ1 RNase‐Free DNase (M6101, Promega, Madison, WI). Absence of DNA was verified via qPCR with *ACTIN* primers (see below) on DNase‐treated RNA samples (−RT control). The purified RNA was synthesized to cDNA using a high‐capacity cDNA reverse transcription kit (Thermofisher, now already mentioned before with Invitrogen). DNase treatment and cDNA synthesis were performed as per the manufacturer's instructions. Twenty microliters of cDNA was diluted with Milli‐Q water to a total volume of 200 μl. The qPCR mix contained 5 μl SYBR‐green (iQ‐SYBR‐green Supermix, Bio‐Rad, Hercules, CA), 0.5 μl forward primer (10 μM), 0.5 μl reverse primer (10 μM), 1.5 μl Milli‐Q water and 2.5 μl cDNA. The qPCR was performed with the following program in a Thermal Cycler (C1000, Bio‐Rad): 95°C for 3 min, then 40 cycles alternating between 95°C for 10 s and 58°C for 30 s. The qPCR was followed by 95°C for 10 s and a melt curve analysis from 65 to 95°C with 0.5°C increments, each step for 10 s. Three biological replicates were used per time point in each treatment and a negative‐control (no cDNA) was done per primer‐pair. The primers for the target genes *StCOL1*, *StSP5G* and *StSP6A* and the reference genes *StEIF3e* and *StACTIN* are given in Fig. S1A (Supporting information) and were previously tested and used by Kloosterman et al. (2013) and Abelenda et al. (2016). In our study, all primers gave a single peak in the melt curve during qPCR, and gel electrophoresis validated that the qPCR amplicons were of the expected sizes (Fig. S1B).

Protein detection was done using western blot analysis. Hundred microliters of extraction buffer [20 mM Tris–HCl, pH 6.8, 8 M urea, 5% sodium dodecyl sulfate (SDS), 15% glycerol, 0.1 mM ethylenediaminetetraacetic acid, 29 mM β‐mercaptoethanol with 2 mM phenylmethylsulfonyl fluoride and 1% protease inhibitor cocktail (Roche, Basel, Switzerland)] was added to approximately 50–100 μg of ground frozen material. After thawing, the samples where vortexed and placed at RT for 5 min after which they were centrifuged at 4°C for 10 min. The supernatant was collected and loaded onto a 10‐well gel (15 μl sample per well in a 10% SDS–polyacrylamide gel or 20 μl sample per well in a Mini‐PROTEAN, 4–20% TGX gel, Bio‐Rad). Protein separation was done with SDS–polyacrylamide gel electrophoresis (SDS–PAGE) in a PowerPac (Basic, Bio‐Rad) with a running buffer (25 mM Tris, 192 mM glycine, 0.1% SDS, pH 8.3) at 100 V and was run until the dye ran out of the gel (approximately 110 min). The gel was then blotted onto a methanol‐activated membrane (Immun‐Blot PVDF, Bio‐Rad). The sandwich containing gel, membrane and filter paper sponges was placed in a transfer buffer (25 mM Tris, 182 mM glycine, 20% SDS, 10% ethanol) and was run for 90 min at 300 mA in the PowerPac. After blotting, the membrane was rinsed in demi water and stained with a Ponceau solution [0.1% (w/v) Ponceau S, 5% (v/v) acetic acid] to obtain a loading control. After staining, the membrane was rinsed in Tris‐buffered saline (TBS; TRIS pH 7.5, 150 mM NaCl) and soaked in a milk‐TBS mix (5% milk powder Elk Campina in TBS) for 1 h. The membrane was then placed in a falcon tube containing 5 ml of 5% milk TBS buffer to which 5 μl of anti‐HA antibody was added [anti‐HA‐peroxidase, high affinity (3F10), Thermofisher] and incubated overnight at 4°C. After incubation, the membrane was washed three times in TBS‐T (30 min total; TBS with 0.05% v/v Tween‐20) and one time for 5 min in TBS. Protein presence was detected using 0.5 ml SuperSignal West Femto Chemiluminescent substrate (Thermofisher) and detected in a chemiluminescence imager (G‐box, Syngene, Bangalore, India).

### Data analysis

The tuberization and flowering times between treatments were compared with a one‐way anova and Fisher's protected lsd (α = 0.05) for pairwise comparisons. These analyses were computed in genstat (18th Edition). Due to confined space in the climate chamber, there were no repetitions of light treatments and only biological replicates were present. Therefore, every plant was considered as an independent experimental unit. The qPCR was performed with three biological replicates per time point and treatment, with the exception of *35S::StCOL1‐HA* samples, which were pooled samples of three plants and had three technical replicates instead. qPCR data were obtained from cfx manager (Bio‐Rad) version 3.1 and expression levels were calculated with 2^−ΔCt^ (Schmittgen and Livak 2008), where ΔCt is the cycle threshold (Ct) difference between the target genes (*StCOL1*, *StSP5G* and *StSP6A*) and the geometric mean of the two reference genes (*EIF3e* and *ACTIN*). For the western blot analysis, one sample was used per time point and treatment. The sample was a pool of leaflets from three plants.

## Results

### The night break at the middle of the dark period had the strongest repressive effect on tuberization

**Figure 3:**
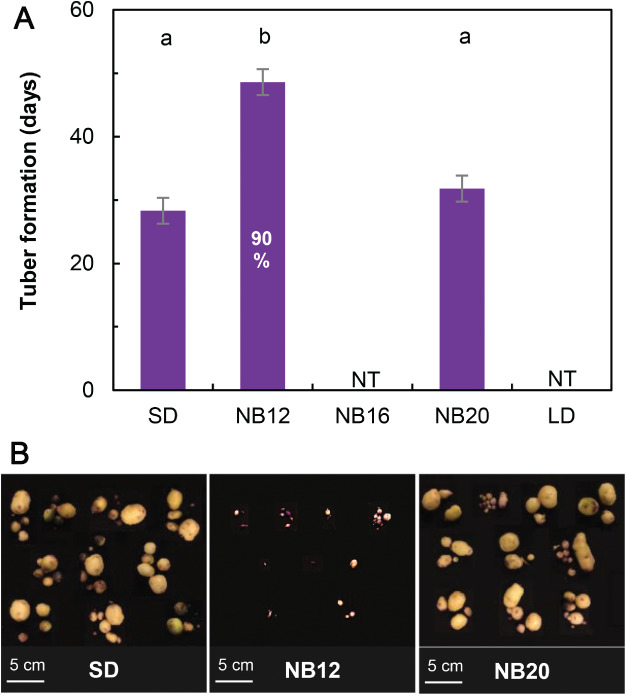
Tuberization in light treatments with or without a night break. *Solanum andigena* plants were grown in short days (SD, 8/16 h light/dark), in short days with night breaks (30 min) applied early in (NB12), at the middle of (NB16) or toward the end of (NB20) every dark period and in long days (LD, 16/8 h light/dark). The numbers of the night break treatments indicate the hours after the start of the light period. (A) Tuberization time in days after transplanting. NT, no tuberization. Significant differences are indicated with letters (α = 0.05, biological replicates, n *=* 10) and error bars indicate the se of difference of the anova. If not all plants tuberized, the percentage that was able to do so is indicated. (B) Tubers at harvest from plants grown in tuber inducing light treatments (8 weeks after transplanting). Plants in NB(16) and LD did not tuberize. The light treatments are described in more detail in Fig. 2.

To determine whether a night break represses tuberization by stabilizing StCOL1 in the dark, night breaks given at different moments during the dark period were tested. In the short‐day treatment, where *S. andigena* was grown under 8 h of light and 16 h of darkness, tuberization occurred after 28 days (Fig. 3A). When a night break was applied in the middle of the dark period (NB16), no tubers were formed at the time of harvest (after 8 weeks). A night break applied early in the dark period (NB12) partially repressed tuberization; tuberization was delayed compared to short days and the tubers were small (Fig. 3). A night break given toward the end of the dark period (NB20) did not delay tuberization time (Fig. 3A). However, tuber biomass was lower than in the short‐day treatment (Table 1). Under long days, no tubers were formed at the time of harvesting, as was expected for *S. andigena*. The time of flower bud appearance was not significantly affected in treatments with a night break compared to the short‐ or long‐day treatments (Fig. S2A). However, the number of leaves formed before the inflorescence did show differences between treatments. Plants grown in the light treatments where no tuberization had occurred (NB16 and LD) also formed more leaves before the inflorescence than the other treatments (Fig. S2B). The flower buds were significantly smaller in the short‐day treatment than in all other treatments (Table 1). Plants that received a night break early in the dark period (NB12) formed the largest flower buds.

**Table 1:** Morphological traits of wild‐type *Solanum andigena* in light treatments with or without a night break. DW, dry weight; LD, long day, 16/8 h light/dark; NB, short days with a night break of 30 min at ZT(12), ZT(16) or ZT(20) (ZT, Zeitgeber time; hours after the start of the light period); SD, short day, 8/16 h light/dark. Significant differences are indicated with letters (α = 0.05, biological replicates, n = 10).

	Tubers #	Tuber DW (g)	Shoot DW (g)	Total DW (g)	Flower bud (mm)
SD	5.6c	1.79c	1.9a	3.7c	1.3a
NB12	2.8b	0.04a	2.9d	2.9b	4.4c
NB16	0a	0a	2.5c	2.5a	2.7b
NB20	5.6c	1.48b	2.2b	3.7c	2.3b
LD	0a	0a	2.8d	2.8b	3.1b

### Coincidence of the light of a night break and *StCOL1* expression does not always lead to an induction of *StSP5G* and repression of *StSP6A* expression

**Figure 4:**
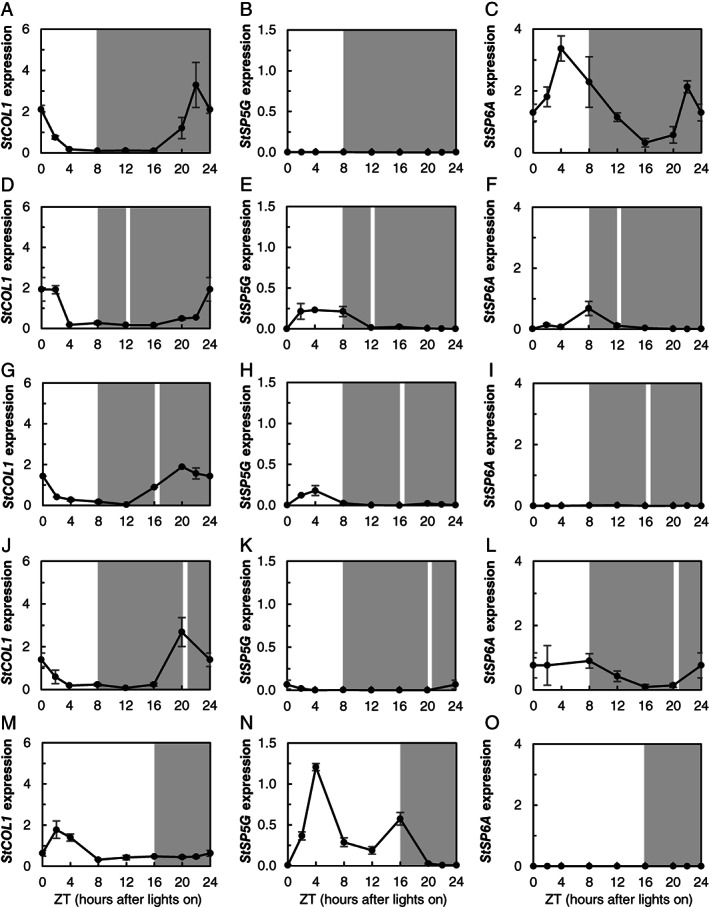
Gene expression in light treatments with or without a night break. *StCOL1*, *StSP5G* and *StSP6A* expression 4 weeks after transplanting in different light treatments in wild‐type *Solanum andigena* plants. Treatments are SD: a short day with 8/16 h light/dark (A–C), NB(12): a short day with a night break at ZT(12) (ZT, Zeitgeber time, hours after lights on) (D–F), NB(16): a short day with a night break at ZT (16) (G–I), NB(20): a short day with a night break at ZT(20) (J–L) and an LD: a long day with 16/8 h light/dark (M–O). The night breaks were applied every night throughout the duration of the experiment. Error bars indicate sem (biological replicates per time point, n = 3).

We hypothesized that a night break given in the middle of the dark period would stabilize StCOL1 and repress tuberization, through induction of *StSP5G* and repression of *StSP6A*. *StCOL1* expression peaked at the end of the dark period in the short‐day treatment (Fig. 4A). In the treatment where the night break was given in the middle of the dark period (NB16), *StCOL1* similarly peaked at the end of the dark period (Fig. 4G). Although the expression pattern was comparable to the short‐day treatment, *StCOL1* was expressed at a low level at ZT(16), the time the night break was applied. In the treatment with the night break given toward the end of the dark period (NB20), the light of the night break coincided with peak expression of *StCOL1* (Fig. 4J), but in the early night‐break treatment (NB12), *StCOL1* expression peaked long after the night break was applied (Fig. 4D). *StSP5G* was not expressed in the short‐day treatment (Fig. 4B). This corresponded to an elevated *StSP6A* expression (Fig. 4C) and the finding that these plants tuberized. In the treatment with the night break in the middle of the dark period (NB16), *StSP5G* was upregulated in the morning, and *StSP6A* was not expressed, which corresponded to the lack of tuberization (Fig. 4H, I). In the late night‐break treatment (NB20), *StSP5G* was hardly upregulated (Fig. 4K), even though the peak of *StCOL1* expression coincided with the light of the night break. *StSP6A* was expressed in this treatment (Fig. 4L), albeit lower than in the short‐day control. *StCOL1* expression in the early night‐break treatment (NB12) did not coincide with the light of the night break, but *StCOL1* expression in the early night‐break treatment was high in the light period at ZT(2), which was not the case in the other treatments. *StSP5G* was induced in the early night‐break treatment (Fig. 4E). However, even though *StSP5G* was expressed higher than the other night‐break treatments, *StSP6A* was still induced (Fig. 4F). The long‐day *StCOL1* expression peaked at ZT(2), and *StSP5G* was highly expressed compared to the other treatments (Fig. 4M,N). As expected, *StSP6A* was not expressed in long days, corresponding to the absence of tuberization (Fig. 4O). Although both the long‐day treatment and the night‐break treatment in the middle of the dark period (NB16) did not tuberize, *StSP5G* expression in the night‐break treatment was very low compared to the long‐day treatment. The expression results show that coincidence of light and *StCOL1* expression does not always lead to an induction of *StSP5G* and strong repression of *StSP6A* and therefore also not to repression of tuberization.

### StCOL1 protein is stabilized during a night break

**Figure 5:**
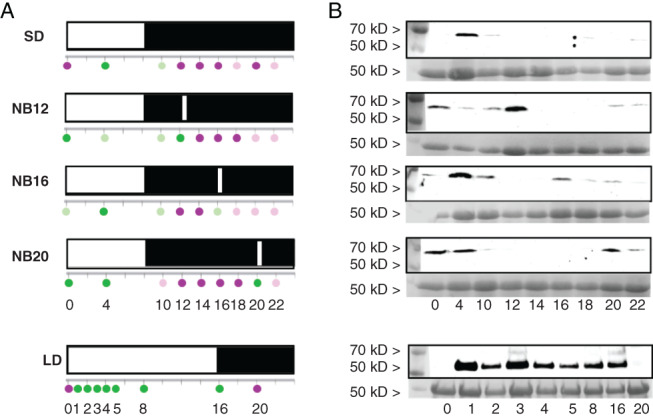
StCOL1 protein in light treatments with or without a night break. Western blot detection of StCOL1 protein in *35S::StCOL1‐HA* plants in an *Solanum andigena* background 4 weeks after transplanting. (A) A schematic representation of protein presence is given at several ZTs (ZT, Zeitgeber time, hours after the start of the light period; magenta, no StCOL1; light magenta, little StCOL1; light green, some StCOL1; green, clear StCOL1 presence). LD, long day; NB, night break; SD, short day. The night breaks were applied every night throughout the duration of the experiment. Numbers behind NB indicate the ZT at which the 30‐min night break is given. (B) Western blot. Protein presence at several ZTs based on a pooled sample of three biological replicates. The sampled time points in the long day differ from the short‐day treatments. A Ponceau stain is given as a loading control (subunit Rubisco approximately 56 kDa; reviewed in Spreitzer 1993).

We expected a night break to repress tuberization by stabilization of StCOL1 protein in the dark period. We determined the presence of StCOL1 protein using western blotting in a line overexpressing StCOL1 (*35S::StCOL1‐HA*). By using an overexpressing line, we were able to observe when the StCOL1 protein was absent and thus determine when it was degraded. By comparing this information with the *StCOL1* expression pattern in wild‐type lines, it could be determined when StCOL1 protein could be present in the leaves. Under short‐day conditions in the overexpressing line, StCOL1 was present in the light and degraded in the dark period (Fig. 5). In all night‐break treatments, StCOL1 was stable during the night break, but was readily degraded as soon as the night break came to an end. StCOL1 appeared to be present in very low levels at the end of the dark period in all treatments. In almost all short‐day treatments, StCOL1 was still vaguely present at the beginning of the dark period (ZT(10)). This was most clear in the treatments with the least tuberization (NB16 and NB12).

### *StSP5G* in plants overexpressing *StCOL1* is similar under long and short days, but *StSP6A* is upregulated in short days

**Figure 6:**
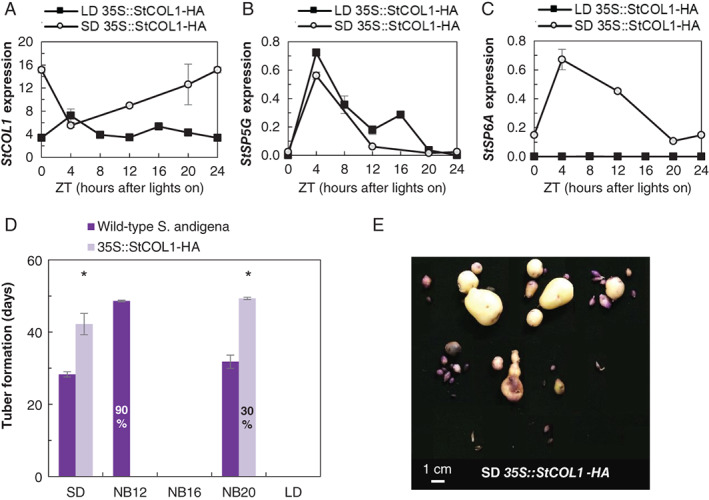
Gene expression and tuberization in *StCOL1‐*overexpressing plants. Gene expression 4 weeks after transplanting and tuberization in *35S::StCOL1‐HA* plants in a *Solanum andigena* background in different light treatments. *StCOL1* (A), *StSP5G* (B) and *StSP6A* (C) expression in short days (SD, open circle) and long days (LD, closed square). n = 3, one pool of three biological replicates with three technical replicates was analyzed per time point. (D) Tuberization in SD, in SD with a night break (NB12, NB16 and NB20) and in LD, in wild‐type *S. andigena* and *35S::StCOL1‐HA* plants. The number behind the NB indicates at what ZT (ZT, Zeitgeber time, hours after the start of the light period) the 30‐min night break was applied. The asterisk indicates a significant difference between the wild type and the overexpressing line (α = 0.05, biological replicates, n *=* 10). If not all plants tuberized, the percentage that was able to tuberize is indicated. Error bars indicate sem. (E) Tubers of *35S::StCOL1‐HA* plants in short days.

The *StCOL1‐*overexpressing line showed a high expression of *StCOL1* throughout the day in both long and short days (Fig. 6A). In both long and short days, *StSP5G* was induced as well (Fig. 6B). However, *StSP6A* expression was only repressed in the long‐day plants (Fig. 6C). In short‐day plants, *StSP6A* was upregulated and tubers were formed (Fig. 6C–E). However, upregulation was lower and tuberization was delayed compared to wild‐type plants (Figs 4C and 6C, D). Tuberization in the *StCOL1‐*overexpressing plants was also delayed in the night‐break treatments. Tuberization in the late night‐break treatment (NB20) occurred more than 2 weeks later than in the wild‐type plants, while tuberization in the early night‐break treatment (NB12) did not occur at all when *StCOL1* was overexpressed (Fig. 6D).

## Discussion

### Repression of tuberization by a night break cannot be explained by coincidence between *StCOL1* expression and light

A night break applied in the middle of the dark period in short days represses tuberization (Batutis and Ewing 1982). Our results show that a night break given in the middle of the dark period suppresses expression of the gene encoding the tuberization signal *StSP6A* and represses tuberization (Figs 3A and 4C). We hypothesized that the functioning of a night break on tuberization could be explained by the coincidence of the light given during the night break, and the high *StCOL1* expression in the dark period. The light of the night break would stabilize StCOL1 protein, which in turn would lead to induction of *StSP5G*, and the repression of *StSP6A* and tuberization. Our results show that *StCOL1* was expressed at a low level during the application of a classic night break in the middle of the dark period (NB16; Fig. 4G). Furthermore, the StCOL1 protein was stable at the time of night‐break application, indicating StCOL1 protein could induce *StSP5G* expression and repress *StSP6A*, which matches our expression results. As suggested by the coincidence model, peak *StCOL1* expression has to coincide with light to induce *StSP5G* and repress tuberization (Imaizumi and Kay 2006, Abelenda et al. 2016), but in the NB16 treatment, peak *StCOL1* expression took place after the night break, in the dark. Thus, coincidence of peak *StCOL1* expression and light cannot explain the functioning of a night break.

An alternative explanation is that light activates PHYB, which is only slowly deactivated by dark reversion (Ruddat et al. 1997) and can remain active to stabilize StCOL1 during peak *StCOL1* expression later in the dark period. PHYB was shown to be involved in StCOL1 stabilization in potato, and in rice‐mediated night‐break inhibition of flowering (Ishikawa et al. 2005, Abelenda et al. 2016). Furthermore, a recent study has shown that night break delayed flowering in tomato is regulated through PHYB (Cao et al. 2018). In the case of a prolonged effect of PHYB‐mediated StCOL1 stabilization, not the coincidence of light and peak *StCOL1* expression, but applying light just before *StCOL1* peak expression is crucial. In the short‐day treatment, light is only applied after the *StCOL1* peak, which may lead to a lack of StCOL1 and thus allow tuberization to occur. In the NB16 treatment, light is applied before the *StCOL1* peak. In this case, the prolonged effect of PHYB may allow for enough StCOL1 to be stabilized, concomitantly with increasing *StCOL1* expression, to repress tuberization. If this hypothesis is correct, we would expect StCOL1 to be present in the period between night‐break application (ZT(16)) and peak *StCOL1* expression (ZT(20); Fig. 4G). However, western blot analysis showed that StCOL1 degradation already took place at ZT(18) (Fig. 5), making it unlikely that enough StCOL1 would be stabilized during peak *StCOL1* expression to repress tuberization. What is even more contradictory to the hypothesis that the coincidence between light and *StCOL1* expression represses tuberization are the unexpected results of the late night‐break treatment NB20. In this treatment, the coincidence of light and peak *StCOL1* expression at ZT(20) did not lead to high *StSP5G* expression or repressed tuberization (Figs 3A and 4J, K), even though StCOL1 protein was present during the night break at ZT(20) (Fig. 5). Plants in the NB20 treatment tuberized just as fast as in the short‐day treatment. These findings do not support the theory that coincidence of light and peak *StCOL1* expression or even application of light just before *StCOL1* expression leads to repression of tuberization. Instead, these results imply that factors other than StCOL1, StSP5G and StSP6A are involved in night‐break repression of tuberization. Expression data from the early night‐break treatment NB12 support this idea. The night break did not coincide with *StCOL1* expression (Fig. 4D), but *StSP5G* was upregulated and *StSP6A* expression was repressed compared to the short day without a night break (Fig. 4E, F). Furthermore, tuberization was severely delayed (Fig. 3A). In tomato, it was shown that night breaks delayed flowering through PHYB‐mediated upregulation of *SP5G* (Cao et al. 2018), which is the tomato ortholog of *StSP5G* (Abelenda et al. 2016, Soyk et al. 2017). It could be the case that *StSP5G* is also induced by PHYB in an early night break, and that this induction happens independent of StCOL1. However, it is surprising that *StSP6A* expression and tuberization are only partially repressed in the early night‐break treatment, considering *StSP5G* expression is higher than in the NB16 treatment, where *StSP6A* and tuberization are fully repressed (Figs 3A and 4E, F, H, I). Although samples for gene expression were taken just before the first tuberization started (28 days), it would be interesting to investigate if *StCOL1* expression changes throughout the development of the plant. Perhaps at an earlier or later developmental stage, coincidence between *StCOL1* expression and light correlates to the tuberization phenotype. However, even if this would be the case, it may not explain why the late night‐break treatment (NB20) tuberized after approximately 32 days, while *StCOL1* peak expression coincided with light only 4 days earlier.

Light‐stabilized StCOL1 during the night cannot explain the repressive function of a night break. The lack of *StSP5G* upregulation despite the coincidence of *StCOL1* and light and the presence of StCOL1 protein at ZT(20) in the NB20 treatment indicates that there is an extra level of control between StCOL1 and *StSP5G* that still needs elucidation or that *StSP5G* is regulated by a transcription factor other than StCOL1. Furthermore, the upregulation of *StSP6A* despite upregulated *StSP5G* in the NB12 treatment suggests that *StSP5G* expression alone does not determine *StSP6A* induction.

### *StCOL1‐*overexpressing plants induce *StSP5G* in both long and short days, but *StSP6A* and tuberization are only induced in short days

In the night‐break treatments, the coincidence of *StCOL1* and light did not always lead to strong repression of *StSP6A* expression and of tuberization. This lack of correlation was also seen in the plants overexpressing *StCOL1*. Although *StCOL1* was expressed throughout the day in both short‐ and long‐day conditions, and *StSP5G* expressed in both day‐lengths, *StSP6A* was only induced in short days. This confirms our earlier finding that upregulation of *StSP5G* does not necessarily lead to repression of *StSP6A*. StSP5G is not a transcription factor and needs an additional factor to affect *StSP6A* expression. Abelenda et al. (2016) suggested that StSP5G represses an inducer of *StSP6A*. An additional control on post‐translational level may determine whether StSP5G is able to repress *StSP6A*. Alternatively, *StSP6A* may not be induced at all in long days, in this case StSP5G may partially repress *StSP6A* in short days, while *StSP6A* is not expressed at all in long days. However, *StCOL1 RNAi* lines with very low *StSP5G* expression had upregulated *StSP6A* in long days (Abelenda et al. 2016), suggesting that *StSP6A* expression is not only activated in short days. Another possibility explaining *StSP6A* expression in *StCOL1‐*overexpressing lines in short days is that another factor that is not repressed by StSP5G induces *StSP6A* in short days. The BEL1‐like transcription factor, StBEL5, is involved in tuberization control in potato (Chen et al. 2003). StBEL5 had been reported to induce *StSP6A* in the leaves and in the right conditions the RNA transcript is transported to the stolons and thought to induce *StSP6A* there (Hannapel et al. 2017). It was found that StBEL5 transcript is stabilized by a polypyrimidine tract‐binding protein (StPTB), which accumulates under short‐day conditions (Banerjee et al. 2006, Cho et al. 2015) and therefore in short days, StBEL5 transcript is increased (Chen et al. 2003). As *StSP6A* expression happens downstream of *StCOL1*, induction of *StSP6A* by StBEL5 in short days may permit some *StSP6A* to be expressed, even when *StCOL1* is overexpressed and *StSP5G* is upregulated. Kloosterman et al. (2013) propose that CYCLING DOF FACTOR 1 (StCDF1), which represses *StCOL1* in short days, may also act directly on *StSP6A* expression. Subsequently, the tuber‐inducing role of StCDF1 by repressing *StCOL1* may be compensated by the overexpression of *StCOL1*, but StCDF1 may also directly activate *StSP6A* in short days. However, if *StSP6A* is upregulated by StCDF1 in short days, it is not clear how a short day with a night break can repress tuberization. Confirming whether *StCDF1* expression is unchanged in night‐break treatments compared to regular short‐day treatments may rule out this possibility.

Our results indicate that additional photoperiodic regulation exists between the StSP5G protein and *StSP6A* expression.

### A night break applied at the middle of the dark period has the strongest repressive effect on tuberization

Before anything was known about the molecular control behind tuberization, a night break was claimed to repress tuberization by dividing a long night into two short nights (Jackson 1999). Our results show that the night break was only able to fully repress tuberization when given at the middle of the night period (NB16). Similar findings were made in the short‐day plants rice and Chrysanthemum, where a night break given in the middle of the night period had the strongest inhibitory effect on the flowering (Horridge and Cockshull 1989, Ishikawa et al. 2005). In long‐day plant wheat, several night breaks were tested and the most successful night break was the one given at the middle of the night period (Pearce et al. 2017). The action of a night break may not be regulated through coincidence between *StCOL1* and light, but through a mechanism involving the night length. However, in our experiments, a different tuberization response was seen in the NB20 and NB12 treatments, which both had the same length of dark periods. The most successful of the two in repressing tuberization (NB12) had a short dark period followed by a night break and a longer dark period. Instead of the length of the dark periods, the time until the first light period may be important for successful repression of tuberization.

### An unknown factor controlled by the duration of the dark period may affect tuberization

Pearce et al. (2017) found the length of darkness before the night break increased the expression of *PPD‐B1*, an allelic variant of *PHOTOPERIOD1* (*PPD1*) whose expression leads to *FLOWERING LOCUS T1* (*FT1*) induction in wheat and may be comparable to *StCOL1* in potato. However, the increase in *PPD‐B1* did not correlate to flowering time, suggesting that other circadian clock genes also play a role in night‐break induced flowering in wheat. Interestingly, more parallels exist between night breaks in wheat and potato. Although *StCOL1* was expressed at a low level during a classic night break, *StSP5G* was induced and tuberization was repressed. In wheat, the greatest night‐break‐mediated flower induction was observed in the middle of the night when *PPD1* expression was low (Pearce et al. 2017). The authors suggested one or more unknown circadian clock genes gated the night‐break effect, which may be the case for potato as well. Recent studies in short‐day Chrysanthemum suggest that flowering depends on the duration of the night. Flowering was only possible when the night‐length exceeded a photosensitive phase for induction of anti‐florigen (Higuchi et al. 2013). The gate for maximal induction of anti‐florigen opens at a constant time after dusk, regardless of the period of light preceding it. Coincidence with light during this period, either when nights are short or when an NB is applied, leads to induction of anti‐florigen. A similar mechanism could take place in potato. An unknown factor may be expressed as soon as the dark period starts, with peak expression taking place in the middle of a long night (ZT16). Coincidence with the light of an NB may lead to the induction of an anti‐tuberigen in potato, which in this case may be *StSP5G*. In long days, peak expression of this unknown factor coincides with the light of dawn, also leading to *StSP5G* induction. An early night break (NB12) may coincide with an early low expression of this unknown gene, while during the late night break (NB20) the photosensitive period is over, explaining the low *StSP5G* expression, even while StCOL1 is stabilized. Although such a mechanism involving an additional factor influenced by the night duration is plausible, not all results can be explained. For instance, why do *StCOL1*‐overexpressing plants have similar high *StSP5G* levels if only in long days the additional factor induces *StSP5G*? Possibly both this factor and StCOL1 act on *StSP5G*. Alternatively, this additional factor may stabilize or activate StSP5G or even repress *StSP6A* independently of StSP5G, explaining why *StSP6A* is fully repressed in long days in *StCOL1‐*overexpressing lines, while in short days some *StSP6A* expression can still occur.

A complex mechanism may take place where multiple factors act on *StSP5G* and *StSP6A*, complicating the existing StCOL1 controlled model explaining potato tuberization. An additional factor that is controlled by the duration of the dark period may be a missing link in the control of photoperiodic tuberization. Three tandemly arranged orthologs of *CO* were identified in potato (*StCOL1*‐*StCOL3*) and it was shown that *StCOL1* is expressed at relatively high levels in leaves, but *StCOL2* levels are much lower and *StCOL3* is almost undetectable (Abelenda et al. 2016). However, expression of *StCOL2* was shown to peak in the middle of a long night, which fits with the expected action period of the additional controlling factor. Additionally, Abelenda et al. (2016) demonstrated that StCOL2 was able to activate *StSP5G* expression. Determining the tuberization phenotype and *StSP5G* and *StSP6A* expression patterns in transgenic plants silenced in *StCOL2* may determine if this paralog of StCOL1 is involved in the regulation of a night break.

## Conclusions

Our results show that coincidence between *StCOL1* expression and light cannot explain why a night break represses tuberization in short days. Night‐break treatments that were given during peak expression of *StCOL1* did not strongly induce *StSP5G* nor substantially repress *StSP6A* expression. Furthermore, plants overexpressing *StCOL1* still expressed *StSP6A* in short days and were able to tuberize, even though *StSP5G* expression was almost identical to expression in long‐day plants, which did not tuberize. Our findings suggest there is an additional level of control between *StCOL1*, *StSP5G* and *StSP6A* expression, which determines whether a plant tuberizes.

## Author contributions

F.D.M.P. was involved in conception and design of study, acquisition of data, analysis and interpretation of data, drafting the article and final approval of the submitted version. E.H. contributed to obtaining of funding, conception of study, critical revision, statistical expertise and final approval of the submitted version. J.A.R. contributed to acquisition of data, interpretation of data, critical revision and final approval of the submitted version. R.G.F.V. contributed to obtaining of funding, critical revision and final approval of the submitted version. C.W.B.B. and L.F.M.M. contributed to obtaining of funding, conception of study, critical revision and final approval of the submitted version.

## Supporting information


**Fig. S1.** Primers used in the study and validation of target gene amplification.
**Fig. S2.** Flowering time in *Solanum andigena* grown in light treatments with or without a night break.
