# Supplementary material for: Coincidence of potato CONSTANS (StCOL1) expression and light cannot explain night‐break repression of tuberization
Source: Physiol Plant. 2018 Dec 21;167(2):250–63. doi: 10.1111/ppl.12885 (PMC7379991; doi:10.1111/ppl.12885)
Supplement: Supplementary file 1 — Fig. S1. Primers used in the study and validation of target gene amplification. Fig. S2. Flowering time in Solanum andigena grown in light treatments with or without a night break. [file PPL-167-250-s001.pdf]

SUPPORTING INFORMATION

A

**Target gene**	**Transcript ID**	**Primer sequences (forward/reverse; 5'-3')**	**Amplicon length (bp)**
StEIF3e (reference)	PGSC0003DMT400076704	F: GGAGCACAGGAGAAGATGAAGGAG<br>R: CGTTGGTGAATGCGGCAGTAGG	164
StACTIN (reference)	PGSC0003DMT400010174	F: GGAAAAGCTTGCCTATGTGG<br>R: CTGCTCCTGGCAGTTTCAA	60
StCOL1	PGSC003DMT400026065	F: GTAGCAACAATTGGGCAAGGG<br>R: AGTAAACGGTACATGTTGCGGA	60
StSP5G	Transcript ID unknown,<br>ITAG: Sotub05g026730.1.1	F: GGTGTGTAGACTTTGGTGTGGTTT<br>R: GGCCTCAAGGCACATCCAT	64
StSP6A	PGSC0003DMT400060057	F: GACGATCTTCGCAACTTTTACA<br>R: CCTCAAGTTAGGGTCGCTTG	75

B

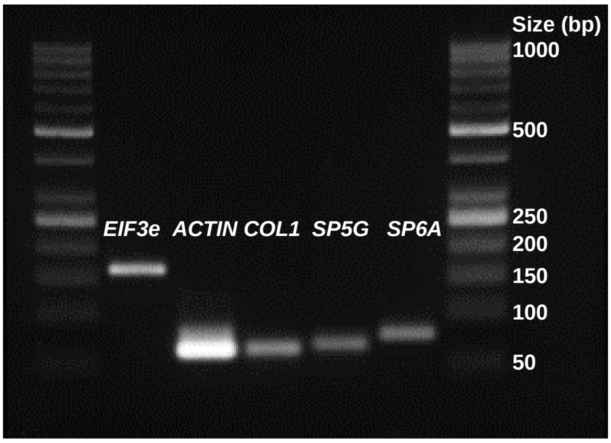

Figure S1. Primer list and verification of qPCR amplicons by agarose gel electrophoresis. (A) Target genes in this study with the corresponding primers used in qPCR and expected amplicon sizes. Primers correspond to the ones used in Kloosterman et al. 2013 and Abelenda et al. 2016 (B) Verification of amplicon sizes from the qPCR by gel electrophoresis. Melt curves during the qPCR showed single peaks for each primer set across all samples. A mixture of four representative samples per primer set was loaded on a 2.5% agarose gel. Amplicon sizes match the expected sizes.

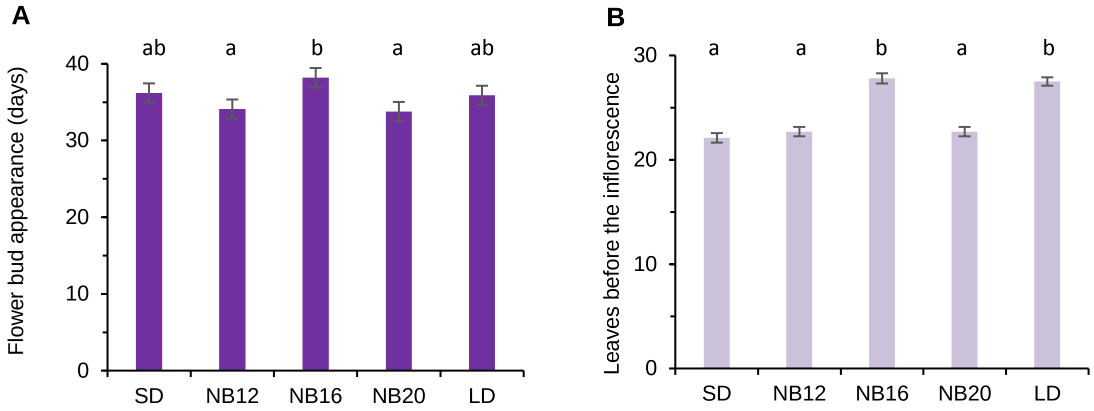

Figure S2. Flowering time of *S. andigena* grown in light treatments with or without a night break. Plants were grown in short days (SD = 8/16 hours light/dark), in short days with night breaks (30 min) applied in the beginning (NB12), in the middle (NB16) or at the end (NB20) of the dark period, and in long days (LD = 16/8 hours light/dark). The night breaks were applied every night for the duration of the experiment. The numbers of the night-break treatments indicate how many hours after the start of the light period the light treatment was given. (A) Flower bud appearance time in days between transplanting and appearance of the first flower bud. (B) Flowering in the number of leaves formed before the inflorescence. Error bars show the standard error of difference of the ANOVA analysis. Significant differences are indicated with letters ($\alpha = 0.05$, biological replicates, $n = 10$).
